# Pretreatment of Mesenchymal Stem Cells Manipulates Their Vasculoprotective Potential While Not Altering Their Homing Within the Injured Gut

**DOI:** 10.1002/stem.2061

**Published:** 2015-06-29

**Authors:** Dean P.J. Kavanagh, Shankar Suresh, Philip N. Newsome, Jon Frampton, Neena Kalia

**Affiliations:** ^1^ Centre for Cardiovascular SciencesUniversity of BirminghamEdgbastonBirminghamUnited Kingdom; ^2^ NIHR Centre for Liver Research and Biomedical Research UnitUniversity of BirminghamEdgbastonBirminghamUnited Kingdom; ^3^ School of Immunity and InfectionUniversity of BirminghamEdgbastonBirminghamUnited Kingdom

**Keywords:** Mesenchymal stem cells, Intravital microscopy, Cell transplantation, Ischemia‐reperfusion injury, Homing, Cell therapy, Inflammation, Chemokines

## Abstract

Mesenchymal stem cells (MSCs) have shown therapeutic promise in many experimental and clinical models of inflammation. However, a commonly reported feature of MSC transplantation is poor homing to injured tissues. Previously, we have shown that pretreatment with cytokines/chemical factors enhances hematopoietic SC adhesion within intestinal microvasculature following ischemia‐reperfusion (IR) injury. Using intravital microscopy, the ability of similar pretreatment strategies to enhance the recruitment of murine MSCs to murine intestinal microvasculature following IR injury was investigated. Primary MSCs were isolated from bone marrow and selected on the basis of platelet‐derived growth factor receptor‐α and SC antigen‐1 positivity (PDGFRα^+^/Sca‐1^+^). MSC recruitment was similar in IR injured gut mucosa when compared with sham operated controls, with limited cell adhesion observed. MSCs appeared contorted in microvessels, suggesting physical entrapment. Although not recruited specifically by injury, MSC administration significantly reduced neutrophil recruitment and improved tissue perfusion in the severely injured jejunum. Vasculoprotective effects were not demonstrated in the lesser injured ileum. Pretreatment of MSCs with tumor necrosis factor (TNF)‐α, CXCL12, interferon (IFN)‐γ, or hydrogen peroxide did not enhance their intestinal recruitment. In fact, TNFα and IFNγ removed the previous therapeutic ability of transplanted MSCs to reduce neutrophil infiltration and improve perfusion in the jejunum. We provide direct evidence that MSCs can rapidly limit leukocyte recruitment and improve tissue perfusion following intestinal IR injury. However, this study also highlights complexities associated with strategies to improve MSC therapeutic efficacy. Future studies using cytokine/chemical pretreatments to enhance MSC recruitment/function require careful consideration and validation to ensure therapeutic function is not impeded. Stem Cells *2015;33:2785–2797*

**Significance Statement**

Little is known about the in vivo kinetics of MSC homing to sites of injury, whether chemical pre‐treatments can modify this event and whether development of such strategies comes at the cost of MSC functionality. In this study, we show that MSC adhesion within IR injured gut mucosa is not only poor, but this phenomenon cannot be enhanced by pre‐treatment with a range of cytokines or chemical factors. However, despite limited homing, MSCs can confer an anti‐inflammatory effect and improve tissue perfusion in some anatomical regions of the gut. However, this novel study highlights a serious consequence of MSC manipulation whereby their therapeutic potential is demonstrated to be abolished following pre‐treatment with particular factors.

## Introduction

Mesenchymal stem cells (MSCs) have rapidly become one of the leading cell therapy candidates for treating a variety of inflammatory and degenerative diseases. Clinically, the preferred route of administration of stem cells (SCs) for cellular therapy is by direct infusion into the bloodstream, which provides a noninvasive delivery method and allows repeated injections of cells 1. Capture of exogenously administered circulating MSCs by injured tissue microvascular endothelium is a prerequisite event for successful therapy, regardless of the mechanisms underlying MSC‐mediated repair 2. Although most of our understanding of MSC‐endothelial interactions comes from in vitro research, limited studies have investigated the actual kinetics of MSC trafficking in vivo at a cellular level. Injected MSCs have been observed intravitally to adhere to healthy mouse ear veins, albeit for short periods of time 3. Vascular entrapment rather than a dependency on active adhesion was further shown as rat MSCs predominantly arrested in healthy cremasteric precapillaries, resulting in cessation of flow in the plugged microvessels 4. In contrast, a recent study demonstrated that active mechanisms, partially dependent on platelets, were involved in governing human MSC adhesion and transmigration within the lipopolysaccharide (LPS)‐stimulated inflamed ear dermis microcirculation 5.

These studies, conducted in vascular beds which are commonly used for intravital observations, provide a glimpse of the kinetics of MSC recruitment in vivo. Since the therapeutic potential of injected MSCs may be directly dependent on their localization, it is anticipated that strategies which enhance local recruitment will improve the effectiveness of cellular therapy through acceleration of tissue recovery 2. We have previously demonstrated that pretreatment of hematopoietic SCs (HSCs) with the reactive oxygen species hydrogen peroxide (H_2_O_2_) or the chemokine stromal derived factor‐1α (CXCL12) enhances their adhesion within the ischemia‐reperfusion (IR) injured gut 6, 7. Nongenetically engineered approaches to enhance HSC delivery offer the potential for clinical use as an adjuvant therapy to increase the effectiveness of HSC therapy. However, it is not known whether MSC recruitment is also an event that can be improved using similar strategies or whether recruitment mediated by injury alone is maximal. Indeed, recent attention has focussed on MSCs primarily due to their anti‐inflammatory and immunomodulatory effects observed in vitro and their low immunogenicity. However, direct evidence of vasculoprotection, and exactly what this comprises, has not been demonstrated acutely in vivo in the presence of disease or a clinically relevant injury.

In this study, we used intravital microscopy, a methodology with single‐cell sensitivity, to first detail the kinetics of MSC homing to IR injured mouse gut. This novel study directly examined MSC homing in a clinically relevant model of injury, which has not previously been performed. MSC therapy has certainly been proposed as a potential treatment for a whole host of ischemic and inflammatory bowel disorders due to their ability to dampen inflammation and promote tissue repair 8. In models of colitis and autoimmune disease of the bowel, MSCs improve a number of markers including diarrhea, body weight and survival 9, 10. Second, we assessed whether our previously successful pretreatment strategies could enhance MSC adhesion with the injured gut. Indeed, suboptimal MSC recruitment has been proposed to explain the apparent differing results between systemically and locally administered MSCs in models of Crohn's disease 11. The ability of tumor necrosis factor (TNF)‐α, H_2_O_2_, CXCL12, and interferon (IFN)‐γ to modify MSC adhesion and therapeutic potential was investigated. These factors were selected based on their well‐established roles in ischemia and inflammation and their documented ability to manipulate the therapeutic behavior of MSCs following administration in hosts 12, 13, 14, 15. During IR injury, TNFα and H_2_O_2_ are released rapidly into the local microenvironment with elevated and sustained concentrations noted as early as the first hour of reperfusion 16, 17. Intestinal IR injury is also associated with increased circulating CXCL12 18 and enhanced IFNγ mRNA expression 19 within 60 and 90 minutes post‐reperfusion respectively.

Although the exact mechanisms that appear to underlie the amelioration of injury by MSCs are unclear, they are thought to involve paracrine release of anti‐inflammatory soluble factors, inhibition of proinflammatory lymphocytes, and induction of T‐cell apoptosis 20. Whether MSCs exert vasculoprotective effects while adherent within the microcirculation of damaged tissue, and how rapidly they do so, is unclear 21, 22. Therefore, the impact of naive or pretreated MSCs on inflammatory neutrophil infiltration was also assessed. The pathophysiology of IR injury is driven, in large, by the activity of neutrophils; these are the major effector cells. Neutrophils are among the first cells to be recruited to IR injured organs, where they release reactive oxygen species, endothelial activators, and proteolytic enzymes. Given the central role of the neutrophil in this injury, it was an appropriate marker to examine the progression of IR injury. Improvements in blood flow to the IR injured gut were also investigated as it is well known that IR injury compromises local tissue perfusion. This is critical to assess as MSCs could inadvertently impact negatively on blood flow by plugging microvessels and thus exacerbate the effects of ischemia.

## Materials and Methods

### Animals

For both intravital microscopy and bone marrow isolation, male C57Bl/6 mice (8–12 week old; Harlan, Oxon, UK) were used for procedures in accordance with the Animals (Scientific Procedures) Act of 1986. For intravital microscopy, anesthetized animals (100 mg/kg ketamine hydrochloride, Zoetis UK, UK; 10 mg/kg xylazine hydrochloride, Chanelle Animal Health, UK; intraperitoneally) underwent tracheotomy and carotid cannulation to facilitate infusion of labeled cells and maintenance anesthetic. IR injury was established through occlusion of the small mesenteric artery using a nontraumatic artery clamp for 45 minutes. Reperfusion was initiated by clamp removal.

### Cells

MSCs were isolated as described previously in detail elsewhere 23. Briefly, muscle and surrounding tissue were removed from the fibulae and tibiae of 8–12 week adult male C57Bl/6 mice. Bones were fractured, marrow contents and remaining bone fragments were incubated with 0.2% collagenase (Wako Chemicals, Osaka, Japan) in Dulbecco's modified Eagle's medium (DMEM) (Sigma‐Aldrich, Poole, UK). Cells were isolated by mechanical dissociation from bone following incubation. Subsequently, MSCs were selected from suspensions using flow cytometry (MoFlo XDP, Beckman Coulter, High Wycombe, UK). Suspensions were labeled with FITC conjugated anti‐mouse Sca‐1 (Ly6A/E; Clone D7, eBioscience, Hatfield, UK), PE conjugated anti‐mouse CD45 (30‐F11; eBioscience, Hatfield, UK), PE conjugated anti‐mouse TER‐119 (Clone TER‐119, eBioscience, Hatfield, UK), and APC conjugated anti‐mouse CD140a (PDGFRα; Clone APA5, eBioscience, Hatfield, UK). Labeled suspensions were washed and resuspended in Hanks’ balanced saline solution (Sigma‐Aldrich) with propidium iodide (eBioscience, Hatfield, UK). Cells were isolated based on positive staining for Sca‐1 and CD140a, along with negative staining for TER‐119, CD45 and PI. This criteria yields an enriched population of proliferative murine MSCs. Isolated cells were maintained in minimum essential medium α (MEMα) supplemented with 10% fetal bovine serum (FBS; Sigma‐Aldrich), l‐glutamine (PAA Laboratories, Yeovil, UK), penicillin/streptomycin (PAA), and 10 ng/ml transforming growth factor‐β (TGFβ; New England Biolabs, Herts, UK). Cells were used for experiments between passages 4 and 9. No correlation between passage number and experimental results were identified within this range.

### Surgical Preparation and Intravital Imaging of the Ileum

Intravital microscopy was used to monitor MSC and neutrophil adhesion in injured intestinal microvasculature 7. Since the mucosal or luminal surface of the intestine is most susceptible to ischemic challenge, the mucosa of the distal ileum was prepared for imaging by cautery incision alongside the anti‐mesenteric border. Exposed mucosal villi were visualized using an inverted intravital microscope (Olympus IX‐81, Olympus, Essex, UK). For analysis, a single ×10 field of view was selected before cell administration. 5 × 10^5^ MSCs, prelabeled with 10 μM 5,6‐carboxyfluorescein diacetate succinimidyl ester (CFDA‐SE, Life Technologies, Paisley, UK), were injected intra‐arterially via the left common carotid at 30 minutes post‐reperfusion and recruitment in the mucosal villi analyzed. Cells were administered at this time point to allow IR injury to be established in the gut. Significant intestinal microcirculatory damage occurs at this point, associated with increased levels of inflammatory factor release. This exposes trafficking MSCs to inflamed microvessels, providing them with an opportunity to adhere rather than pass through the gut. Some MSCs were pretreated in a 1‐ml suspension with 100 ng/ml TNFα (Peprotech, London), 100 ng/ml interleukin (IL)−1β (Peprotech, London), 100 ng/ml CXCL12 (Peprotech, London), or 100 µM H_2_O_2_ (Sigma‐Aldrich) for 1 hour before their systemic administration. Treatments were terminated by excess media and centrifugation. Pellets were resuspended in 100 µl of saline before infusion. Digital videos were recorded for 1 minute, every 5 minutes, for an hour post‐reperfusion. Adherent cells were identified as those that remained stationary for ≥30 seconds. Images were also obtained post‐mortem from the serosal surface of the ileum, mucosal, and serosal surfaces of the proximal jejunum, liver, spleen, and lungs. To monitor neutrophil recruitment, mice were subjected to 45 minutes ischemia followed by 240 minutes reperfusion. Mice received an intra‐arterial injection of 5 μg anti‐mouse PE‐Gr‐1 antibody at 5 minutes and 235 minutes post‐reperfusion (RB6–8C5; eBioscience, Hatfield, UK). This dose has been shown previously to efficiently label neutrophils while not altering functional behavior 24. At 240 minutes post‐reperfusion, the mucosal villi of the more proximal jejunal region of the small intestinal and the terminal ileum were prepared for intravital imaging. Videos for analysis were obtained from five regions in each anatomical area (ileum/jejunum). Data were stored digitally and analyzed off‐line (Slidebook, Intelligent Imaging Innovations, Denver, CO, USA).

### Blood Flow Analysis

Mucosal blood flow was monitored using single channel laser Doppler flowmetry (moorVMS‐LDF, Moor Instruments, Devon, UK). Three flux values were taken from independent areas in either the jejunum or ileum. All data were normalized to preischemic flux and presented as a flux ratio.

### Static Protein Substrate Adhesion Assay

The 96‐well plates (Nunc, Rochester, NY, USA) were coated by incubation with 10 μg/ml recombinant murine (rm) Intercellular Adhesion Molecule 1 (rmICAM‐1), 10 μg/ml vascular cell adhesion protein 1 (rmVCAM‐1), or 10 μg/ml mucosal vascular addressin cell adhesion molecule 1 (rmMAdCAM‐1) (R&D Systems, Abingdon, UK) at room temperature (RT) for 1 hour. Wells were washed and the plate blocked using 10 mg/ml heat denatured bovine serum albumin (BSA) (Sigma‐Aldrich) at RT for 1 hour. MSCs were labeled with 10 μM CFDA‐SE to enable visualization. Subsequently, cells were pretreated with 100 μM H_2_O_2_, 100 ng/ml CXCL12, 100 ng/ml TNFα, or 100 ng/ml IFNγ for one hour. Wells were washed and pretreated MSCs were incubated with coated plates for 20 minutes at RT. Following incubation, cells were fixed to proteins using 2% glutaraldehyde (Sigma‐Aldrich) for 15 minutes at 37°C. Wells were washed and imaged using an EVOS digital inverted fluorescent microscope and GFP light cube (both Life Technologies). Cell adhesion was quantitated and expressed as a ratio against cell adhesion on BSA following identical treatments.

### Static Endothelial Cell Adhesion Assays

Immortalized colonic endothelial cells (ECs) were obtained as described previously 25 from the Immortomouse (Charles River, MA, US). ECs were expanded at 33°C in MEM D‐valine (US Biological, TX, US), supplemented with 10% FBS (Sigma‐Aldrich), l‐glutamine (Sigma‐Aldrich), Penicillin/Streptomycin (Sigma‐Aldrich), 1% vitamin mix (Sigma‐Aldrich), 1% nonessential amino acids (Sigma‐Aldrich), and 10 U/ml IFNγ (Peprotech). ECs were cultured for experiments at 37°C in DMEM containing 10% FBS, l‐glutamine and Penicillin/Streptomycin. For experiments, ECs were grown to confluence in gelatin‐coated 24 well plates (Nunc). To activate the endothelium, 100 ng/ml TNFα (Peprotech) was added at 37°C for 4 hours. MSCs were pretreated with 100 μM H_2_O_2_, 100 ng/ml CXCL12, 100 ng/ml TNFα, or 100 ng/ml IFNγ for 1 hour. Following pretreatment, MSCs were incubated with ECs at 37°C for 20 minutes. Nonadherent cells were washed and wells were fixed with 10% formalin for 15 minutes at 37°C and imaged using an EVOS FL digital‐inverted fluorescent microscope.

### Enzyme Linked Immunosorbent Assay

The release of potentially active factors (both pro‐ and anti‐inflammatory) from MSCs was tested using commercially available ELISAs (eBioscience, Hatfield, UK). PDGFRα+ MSCs were grown to 90% confluence in 24‐well plates. MSCs were subsequently treated for 24 hours with either 100 ng/ml TNFα, 100 ng/ml IFNγ, 100 ng/ml CXCL12, or 100 µM H_2_O_2_ and their supernatants obtained. These supernatants were analyzed by ELISA for IL‐1β, TNF‐α, IL‐10, IL‐13, and IL‐6. Assays were performed according to manufacturer's instructions. Dynamics of IL‐6 release was examined in detail following treatment with 100 ng/ml TNFα or 100 ng/ml IFNγ. After treatment for 1 hour, supernatant was removed and labeled H1. The same MSCs were treated further for an additional hour in αMEM containing 100 ng/m TNFα or 100 ng/ml IFNγ. This supernatant was isolated, labeled H2 and fresh media containing TNFα or IFNγ added. This culturing routine continued for 5 hours (sample H5). Supernatants were filtered and stored at −20°C until analyzed.

### Statistical Analysis

Intravital microscopy and laser Doppler data were analyzed by two‐way analysis of variance (ANOVA) followed by comparison corrected post‐tests to identify points of significance. Other multiple comparisons were analyzed by one‐way ANOVA followed by comparison corrected post‐hoc tests. Direct comparison of two groups was performed by unpaired Student's *t*‐test. Data are presented as mean ± SEM.

## Results

### Increased Adhesion of Primary PDGFRα^+^ MSCs Is Not Observed Following Intestinal IR Injury

**Figure 1:**
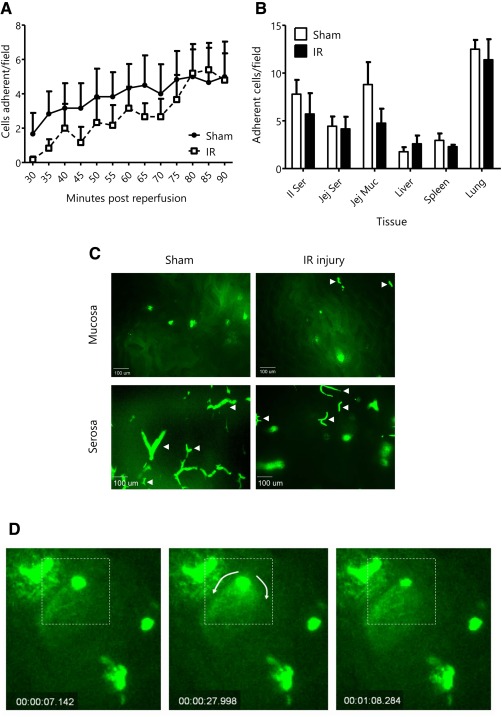
Mesenchymal stem cells (MSCs) are not recruited specifically as a result of injury and appear contorted in the vasculature. **(A)**: MSC recruitment to ischemia‐reperfusion (IR) injured ileum was not significantly enhanced compared with sham operated controls (mean cells adherent/field ± SEM; *n* ≥ 5). **(B)**: MSC recruitment was not enhanced in other tissues as a result of intestinal IR when compared with sham controls (mean cells/field ± SEM; *n* ≥ 5). **(C)**: Representative images are shown of MSCs present in the lumenal mucosa and outer serosal wall following sham or IR injury. MSCs appeared elongated and contorted primarily in the serosal microcirculation (white arrows). **(D)**: Occassionally, MSCs were observed appearing to “release” intracellular contents into the microvasculature. Abbreviation: IR, ischemia‐reperfusion.

MSC adhesion within the mucosal microcirculation of the ileum was not enhanced in IR injured animals and was no different to that observed in sham mice (Fig. 1A, 1C). Indeed, numbers of adherent cells were low (between 2 and 4 cells per field of view) in both sham and injured mice, albeit increasing gradually over the course of the experiment. Adhesion was primarily “first pass”; few MSCs were observed trafficking through the intestine during the remainder of the experiment. Microscopic post‐mortem examination of additional sites in the intestine and other organs revealed that recruitment was not enhanced in remote organs as a result of intestinal injury (Fig. 1B). Unsurprisingly, the highest presence of cells was observed in the pulmonary capillaries in both sham and injured mice (Fig. 1B). The majority of adherent SCs in the mucosal microcirculation appeared smaller and rounded in shape, in contrast to those in the outer serosal layer where MSCs primarily displayed an elongated and more contorted shape. These appearances were characteristic of vascular plugging by MSCs (Fig. 1C). Interestingly, MSCs adherent within the mucosal microcirculation of injured mice occasionally appeared to spontaneously release contents, evidenced by extrusion of fluorescent content and then decreasing in size (Fig. 1D).

### MSCs Reduce Neutrophil Recruitment and Improve Tissue Blood Flow in IR Injured Jejunum

**Figure 2:**
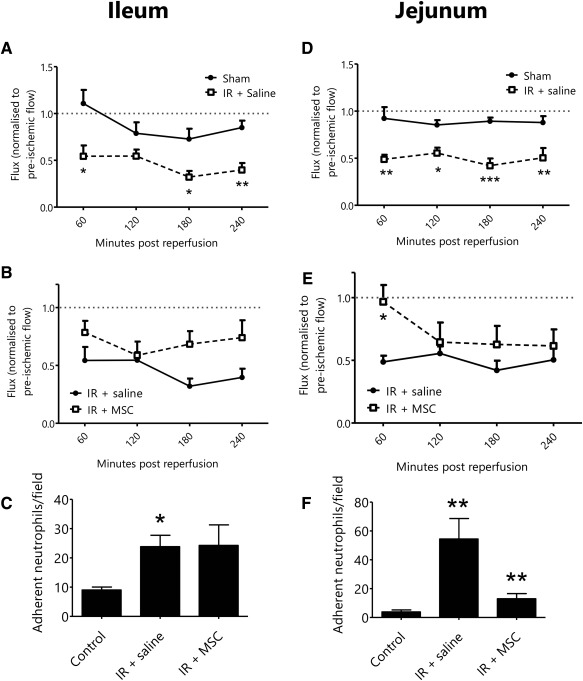
Mesenchymal stem cells (MSCs) improve tissue blood flow and reduce neutrophil recruitment in the severely injured jejunum. **(A)**: Blood flow, as measured by laser Doppler, was significantly reduced following ischemia‐reperfusion (IR) injury when compared with sham mice (IR + S: IR injury with saline bolus; results represent normalized flux ± SEM; *n* ≥ 5). **(B)**: Blood flow was not significantly improved in the ileum by administration of MSCs when compared with saline treated controls (normalized flux ± SEM; *n* = 6). **(C)**: Neutrophil recruitment was significantly increased in the ileum following IR injury. Administration of MSCs did not reduce neutrophil recruitment following IR (mean adherent neutrophils/field ± SEM; *n* = 5). **(D)**: Blood flow was significantly reduced in the jejunum following IR injury when compared with sham controls (normalized flux ± SEM; *n* = 6). **(E)**: Jejunal blood flow was significantly improved at the earliest timepoint in mice receiving MSCs (normalized flux ± SEM; *n* = 6). **(F)**: Neutrophil recruitment was increased in the jejunum following IR injury with MSCs downregulating their adhesion (mean adherent neutrophils/field ± SEM; *n* = 5). Abbreviations: IR, ischemia‐reperfusion, MSC, mesenchymal stem cell.

**Figure 3:**
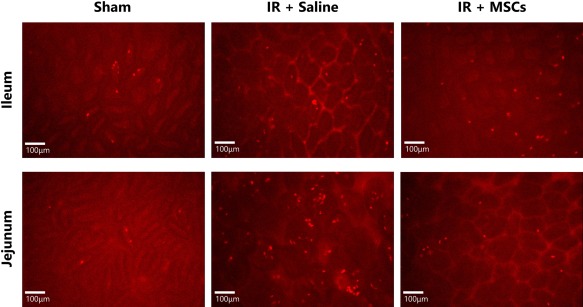
Neutrophil recruitment following ischemia‐reperfusion (IR) injury with or without administration of mesenchymal stem cells (MSCs). Neutrophils were labeled in vivo and their recruitment monitored in the microvasculature. Representative images are shown of the ileal and jejunal mucosa of mice following sham injury, IR injury with a saline bolus (IR + Saline) or IR injury with administration of MSCs (IR + MSC). Abbreviations: IR, ischemia‐reperfusion, MSC, mesenchymal stem cell.

Our previous observations identified that intestinal IR injury was associated with a difference in the degree of macroscopic injury in the proximal jejunum and the terminal ileum, with the former appearing hemorrhagic, severely congested and swollen 26. As varying macroscopic injury occurs in these regions, both were assessed to determine whether blood flow and neutrophil adhesion could be modified by local MSC presence. First, we showed that ileal blood flow was significantly reduced following IR injury when compared with sham blood flow and failed to reach preischemic levels (e.g., normalized flux at 240 minutes post reperfusion: sham: 0.85 ± 0.07 vs. IR: 0.40 ± 0.07; *p* < 0.01; Fig. 2A). Although ileal blood flow appeared to be improved in IR injured mice receiving MSCs, this difference was not significant when compared with mice not receiving MSCs (Fig. 2B). Neutrophil adhesion in IR injured ileal mucosa was significantly higher than that identified in sham controls (adherent neutrophils/field: control: 9.0 ± 1.0 vs. IR: 23.8 ± 3.9; *p* < 0.05) and was not reduced by the administration of MSCs (Fig. 2C, 3).

Tissue blood flow in the jejunum was also significantly reduced following IR injury and, similarly to the ileum, failed to restore to preischemic levels (e.g., normalized flux at 240 minutes post reperfusion: sham: 0.87 ± 0.07 vs. IR: 0.50 ± 0.10; *p* < 0.01; Fig. 2D). However, blood flow was significantly improved at 60 minutes post‐reperfusion in mice receiving MSCs (normalized flux at 60 minutes reperfusion: IR: 0.49 ± 0.05 vs. IR + MSCs: 0.97 ± 0.13; *p* < 0.05; Fig. 2E). This beneficial effect was transient and not observed at 4 hours post‐reperfusion (normalized flux at 240 minutes reperfusion: IR: 0.50 ± 0.10 vs. IR + MSC: 0.61 ± 0.13). Neutrophil adhesion in IR injured jejunum was also significantly increased when compared with sham controls (adherent neutrophils/field: control: 3.8 ± 1.3 vs. IR: 54.4 ± 14.2; *p* < 0.01; Figs. 2F, 3). The greater susceptibility of the jejunum to injury was further reflected by higher levels of neutrophils adherent within IR injured jejunal mucosal microcirculation (54.4 ± 14.2; >14× that in shams) compared with the ileum (23.8 ± 3.9; >2.5× that in sham). However, in the jejunum, neutrophil recruitment was significantly reduced in IR mice receiving MSCs (adherent neutrophils/field: IR: 54.4 ± 14.2 vs. IR + MSCs: 13.0 ± 3.6; *p* < 0.01; Fig. 2F).

### Pretreatment of MSCs Did Not Enhance Their Adhesion

**Figure 4:**
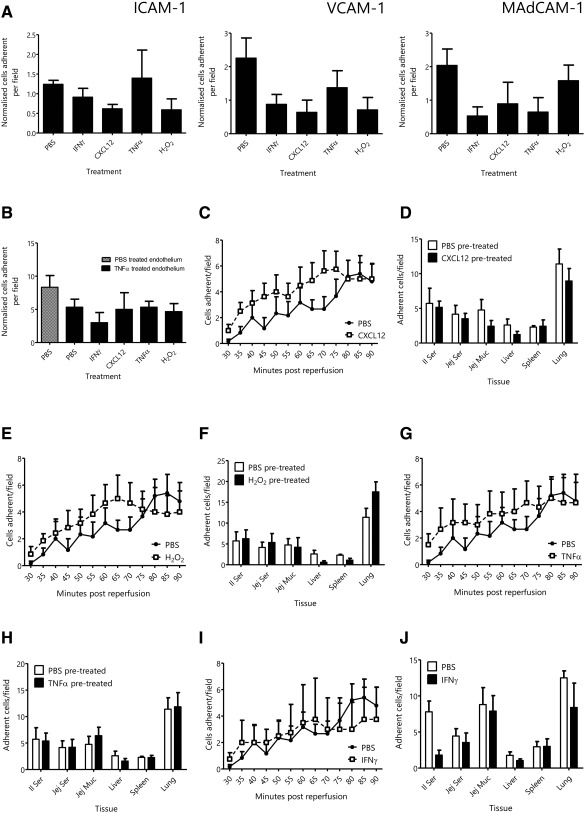
Pretreatment of mesenchymal stem cells (MSCs) with CXCL12, hydrogen peroxide (H_2_O_2_), tumor necrosis factor (TNF)‐α, or interferon (IFN)‐γ did not enhance MSC adhesion both in vitro and in vivo. **(A)**: Treatment of MSCs with a variety of agents did not enhance their ability to adhere to the immobilized protein substrates ICAM‐1, VCAM‐1, or MAdCAM‐1. **(B)**: Similarly, MSC adhesion to murine colonic endothelium was not enhanced by pretreatment. Interestingly, MSC adhesion was not dependent on the activation state of the endothelium. **(C, E, G, I)**: Treatment of MSCs with CXCL12, H_2_O_2_, TNFα, or IFNγ did not significantly enhance their recruitment in the ileum following ischemia‐reperfusion (IR) injury. **(D, F, H, J)**: Additionally, pretreatment of MSCs did not affect their tissue distribution in the IR mouse following administration. Abbreviations: H_2_O_2_, hydrogen peroxide; ICAM, Intercellular Adhesion Molecule 1; IFN‐γ, interferon‐γ; MAdCAM‐1, mucosal vascular addressin cell adhesion molecule 1; PBS, phosphate‐buffered saline; TNF‐α, tumor necrosis factor‐α; VCAM, vascular cell adhesion protein 1.

Pretreatment of MSCs with CXCL12, H_2_O_2_, TNFα, or IFNγ did not enhance their adhesion to immobilized endothelial ligands ICAM‐1, VCAM‐1, or MAdCAM‐1 (Fig. 4A) or to murine colonic endothelium (Fig. 4B) when assessed using static in vitro adhesion assays. Similarly, no pretreatment strategy increased MSC adhesion in vivo in the ileum following IR injury or in any additional organs when compared with phosphate‐buffered saline (PBS)‐treated control cells (Fig. 4C–4J).

### TNFα and IFNγ Pretreatment Elicits a Rapid Release of IL‐6 from MSCs

**Figure 5:**
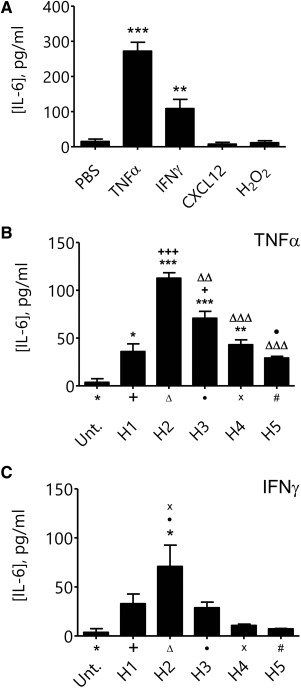
Mesenchymal stem cells (MSCs) rapidly release interleukin (IL)−6 following stimulation with tumor necrosis factor (TNF)‐α or interferon (IFN)‐γ. **(A)**: MSCs release IL‐6 following 24 hours treatment with TNFα or IFNγ, but not CXCL12 or hydrogen peroxide (mean [IL‐6], pg/ml ± SEM; *n* = 4). **(B)**: The supernatants of TNFα stimulated MSCs were isolated and examined at hourly intervals; the media used to replace the isolated supernatants contained fresh TNFα. Release of IL‐6 begins early and peaks in sample H_2_, which represents the second hour of MSC treatment with TNFα (mean [IL‐6] ± SEM; *n* = 3). **(C)**: Similarly, following treatment with IFNγ, IL‐6 release peaks in sample H2 (mean [IL‐6] ± SEM; *n* = 3). For panels (B) and (C), *, versus untreated; +, versus H1; Δ, versus H2; •, versus H3; x, versus H4. Abbreviations: IFN‐γ, interferon‐γ; IL‐6, interleukin‐6; PBS, phosphate‐buffered saline; TNF‐α, tumor necrosis factor‐α.

MSCs were treated with 100 ng/ml CXCL12, 100 µM H_2_O_2_, 100 ng/ml TNFα, or 100 ng/ml IFNγ for 24 hours and the resulting supernatant was analyzed using ELISAs for pro‐ and anti‐inflammatory factors. IL‐10, IL‐13, IL‐1β, and TNFα release was not detected with any of the pretreatment strategies (data not shown). However, both TNFα and IFNγ pretreatment induced significant release of IL‐6 into the supernatant (PBS: 15.2 ± 6.7 g/ml; TNFα: 272.3 ± 25.03 pg/ml (*p* < 0.001 vs. PBS); and IFNγ: 108.9 ± 26.1 pg/ml (*p* < 0.01 vs. PBS); Fig. 5A). The dynamics of IL‐6 with these two pretreatments was further examined in detail. Upon stimulation with TNFα, significant levels of IL‐6 were released that peaked at 2 hours, that is, in sample H2, with decreasing but significant detection observed thereafter in H3 and H4 samples (Fig. 5B). The pattern of IL‐6 release, although broadly similar with IFNγ, was generally lower than release induced by TNFα, and only significantly raised at H2 (Fig. 5C).

### Pretreatment of MSCs with TNFα Abolishes Their Vasculoprotective Effects In Vivo

**Figure 6:**
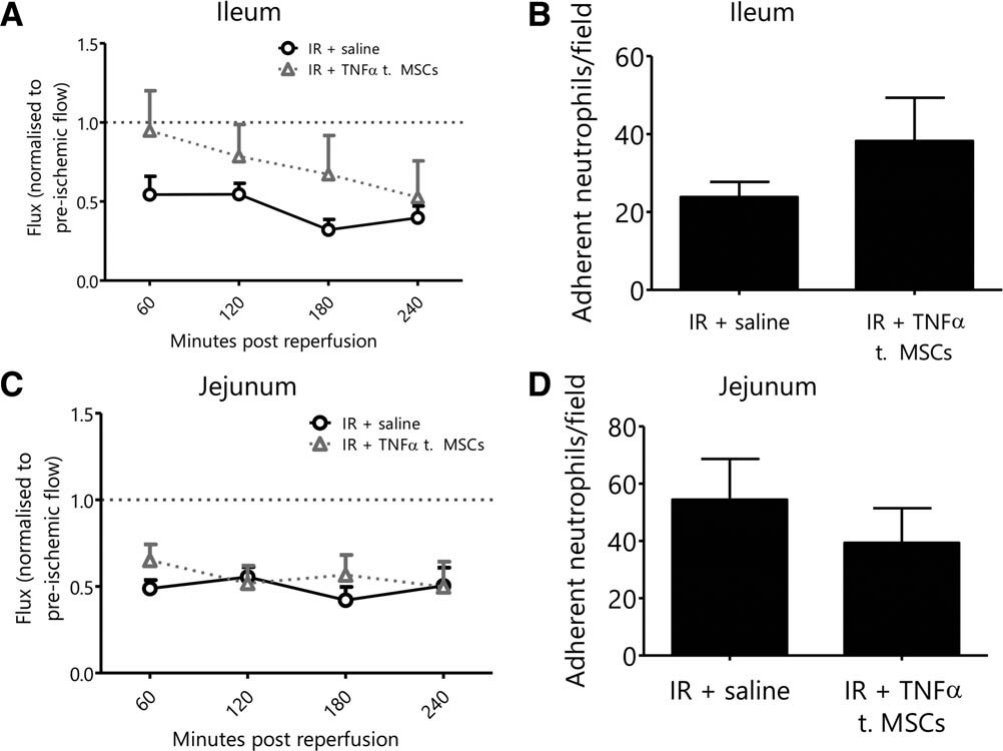
Pretreatment of mesenchymal stem cells (MSCs) with TNFα abolishes their vasculoprotective effects in vivo. **(A)**: Administration of tumor necrosis factor (TNF)‐α treated MSCs did not improve ileal blood flow following ischemia‐reperfusion (IR) injury (normalized flux ± SEM, *n* ≥ 4). **(B)**: Administration of TNFα treated MSCs did not reduce neutrophil recruitment in the ileum following IR injury when compared with mice receiving a saline bolus (mean adherent neutrophils/field ± SEM; *n* = 5). **(C)**: Similarly, administration of TNFα treated MSCs did not improve jejunal blood flow following IR injury (normalized flux ± SEM, *n* ≥ 4). **(D)**: Administration of TNFα treated MSCs did not reduce neutrophil recruitment in the jejunum following IR injury when compared with mice receiving a saline bolus (mean adherent neutrophils/field ± SEM; *n* = 5). Abbreviations: IR, ischemia‐reperfusion, MSC, mesenchymal stem cell; TNF‐α, tumor necrosis factor‐α.

Having demonstrated that TNFα and IFNγ were most potent at stimulating release of IL‐6 from MSCs, their ability to elicit anti‐inflammatory and vasculoprotective effects in the ileum and jejunum in vivo was assessed intravitally. Administration of TNFα prestimulated MSCs did not improve ileal tissue blood flow when compared with mice receiving nonstimulated MSCs (Fig. 6A). No reduction in neutrophil adhesion was observed in the ileum at 4 hours post‐reperfusion (Fig. 6B). As shown previously, administration of unstimulated MSCs improved blood flow at early time points in the severely damaged jejunum and significantly reduced neutrophil adhesion (Fig. 3F, 3G). However, this previously vasculoprotective effect was lost when TNFα‐stimulated MSCs were used (Fig. 6C, 6D).

### Pretreatment of MSCs with IFNγ Either Renders MSCs Vasculoprotective in Areas of Limited Injury or Abolishes This Effect in Severely Damaged Areas In Vivo

**Figure 7:**
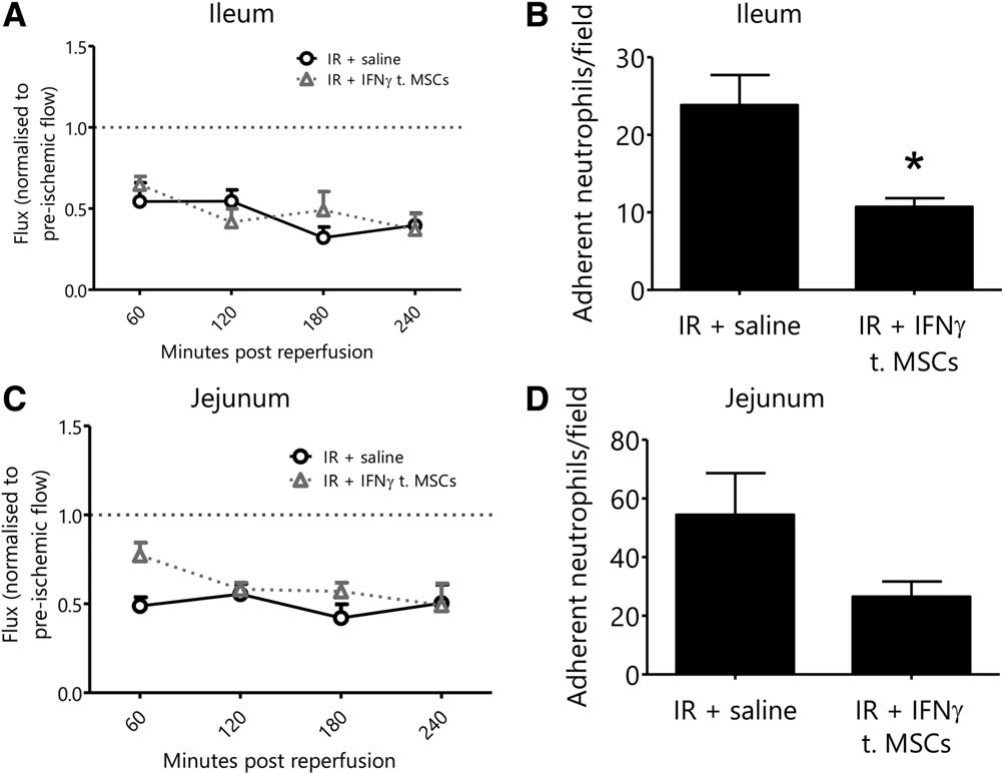
Pretreatment of mesenchymal stem cells (MSCs) with interferon (IFN)‐γ renders MSCs vasculoprotective in areas of limited injury. **(A)**: Administration of interferon (IFN)‐γ treated MSCs did not improve ileal blood flow following ischemia‐reperfusion (IR) injury (normalized flux ± SEM, *n* ≥ 4). **(B)**: Administration of IFNγ‐treated MSCs reduced neutrophil recruitment in the ileum following IR injury when compared with mice receiving a saline bolus (mean adherent neutrophils/field ± SEM; *n* = 5). **(C)**: Similarly, administration of IFNγ treated MSCs did not improve jejunal blood flow following IR injury (normalized flux ± SEM, *n* ≥ 4). **(D)**: Administration of IFNγ treated MSCs did not reduce neutrophil recruitment in the jejunum following IR injury when compared with mice receiving a saline bolus (mean adherent neutrophils/field ± SEM; *n* = 5). Abbreviations: IFN‐γ, interferon‐γ; IR, ischemia‐reperfusion, MSC, mesenchymal stem cell.

Administration of IFNγ‐stimulated MSCs did not improve ileal tissue blood flow compared with mice receiving nonstimulated MSCs (Fig. 7A). As shown earlier, administration of unstimulated MSCs did not reduce neutrophil adhesion in the IR injured ileum (Fig. 6B). However, administration of IFNγ‐stimulated MSCs significantly (*p* < 0.05) reduced neutrophil recruitment in the lesser injured ileum following IR injury (Fig. 7B). Again the previously vasculoprotective effects of unstimulated MSCs in the injured jejunum was lost when IFNγ‐stimulated MSCs were used (Fig. 7C, 7D).

## Discussion

Cell‐based therapies, including those using MSCs, are limited by inefficient homing and capture of cells by injured tissue microcirculation. Therefore, it is accepted that enhancing their adhesion following systemic delivery may improve therapeutic efficacy. However, no studies have directly tracked the homing of MSCs within a clinically relevant model of injury at a cellular level. Furthermore, the acute effects of MSCs within their immediate vascular environment, which may mechanistically explain their potential therapeutic efficacy, have not been directly monitored. In this study, we provide evidence that very few injected MSCs actually home to and are retained within IR injured gut mucosa, with no differences observed between healthy and injured tissue. This is in contrast to our extensive studies on HSCs in which a four‐ to fivefold higher number of adherent cells were observed within a similarly injured organ 7, 27. MSC adhesion was not enhanced using pretreatment strategies shown previously by us and others to increase HSC adhesion. However, despite this, vasculoprotection was afforded by recruited MSCs, but this was dependent on the severity of the injury and degree of inflammation. What was most striking was the observation that pretreatment strategies rendered potentially therapeutic cells relatively incompetent. This clearly has important consequences when designing protocols for clinical translation, particularly in the context of intestinal IR injury.

Although MSC homing has been assessed using various noninvasive techniques, including whole animal IVIS, x‐ray, ultrasound and MRI, these methods do not have single cell resolution. Therefore, the actual number of SCs homing is likely underestimated or unidentified. Our more sensitive approach demonstrated that limited cells freely circulated through the mucosal microcirculation and of these, few became adherent. This lack of a firm interaction with mucosal endothelium may be due to large MSCs (up to 25 μm 28) becoming trapped upstream before entering the small diameter mucosal capillaries (9–12 μm 29). This was evidenced by the striking appearance of elongated MSCs observed in the outer wall serosal microvessels. Indeed, a number of distorted MSCs were frequently observed to line the length of a serosal microvessel. The phenotype of the MSCs in the serosa also suggests only smaller or possibly more deformable MSCs were delivered to the mucosa, as evidenced by the round physical phenotype of cells identified in this region. Work by Toma and colleagues also illustrated the difficulty faced by large MSCs trafficking through microvessels 4. They noted microvascular plugging in cremaster muscle following MSC transplantation. Interestingly, the authors also noted that while mononuclear cells (MNCs) could migrate through membranes with a pore size of 5 μm, MSCs struggled to migrate through pores of 10 μm 4 This did not appear to be due to poor deformability of MSCs as the authors provided evidence that MSCs and MNCs were equally deformable, leading them to suggest that MSC entrapment was primarily a factor of their physical size 4

The lack of circulating MSCs entering the intestinal mucosa is further compounded by the anatomy of the gut wall vasculature. Mesenteric vessels enter the intestine via the outer serosa with parallel branches supplying the serosa, muscle, and submucosal layers. Each mucosal villus is supplied by a central arteriole which originates at right angles to the parent submucosal arteriole. Although the majority of cellular components including neutrophils within the submucosal arteriole continue to the mucosa, this angle would make it difficult for large MSCs to gain similar access. Although decreasing blood flow rates, or even stopping flow completely, has been shown to be conducive (if not necessary) for MSC adhesion in vitro 30, 31, no increased adhesion was observed in vivo despite a decrease in mucosal blood flow in the IR injured gut. Reducing blood flow may mean less MSCs are delivered to injured tissue. However, we have previously demonstrated that HSCs (and indeed inflammatory leukocytes) are able to adhere in increased number despite a reduction in intestinal blood flow. It is also possible that vasoconstriction in serosal arterioles post‐reperfusion at the time of MSC infusion may limit or restrict the ease with which injected MSCs can flow to the mucosa 32.

It is difficult to say definitively that intestinal MSC recruitment is driven purely by their size. Although physical entrapment is certainly a major mechanism governing local MSC presence, active mechanisms may also be involved as MSCs express a wide range of adhesion molecules (l‐selectin, CD44, CD49a‐f, CD29, CD18, among others) which have been shown to be important in MSC homing 21, 22. Indeed, we have previously shown that if MSC adhesion increases with injury, for example, in the liver, integrins and nonintegrins can mediate their recruitment 30. Given that adhesion in our model was not increased following injury, we propose that the presence of MSCs in the injured intestine is mediated (at least primarily) by passive entrapment and that active, molecular driven recruitment may not play a significant role in this model. This is in contrast to our previous studies in which enhanced active HSC recruitment was observed in the gut following a similar IR injury 7. HSCs are much smaller, with primitive HSCs possessing diameters of around 9.5 µm 33. As such, they can most likely circulate relatively unhindered through the intestinal microcirculation, making frequent interactions with inflamed endothelium. This increases the likelihood of firm HSC‐endothelial interactions taking place, which may explain why an increased HSC presence is observed in IR injured gut compared with sham. In addition, preliminary atomic force microscopy studies in our laboratory suggest MSCs are significantly less deformable than HSCs, when comparing biomechanical properties such as rupture forces (Du M, unpublished observations).

Despite small numbers of MSCs being recruited to injured mucosa, significant down regulation of neutrophil recruitment and improvements in tissue perfusion were observed. This is the first study to directly demonstrate and specify the vasculoprotective effects that MSCs can confer in vivo and the speed at which these events take place post‐MSC infusion. Interestingly, the capacity for MSCs to attenuate injury varied between different anatomical regions of the intestine and appeared to be dependent on the degree of injury. Grossly visible damage was apparent in the jejunum, with neutrophil adhesion more than doubled in this region compared with the ileum. Varying susceptibility of the gut to IR injury has been described previously by us 26 and also Chan et al., who demonstrated that basal levels of protective nitric oxide (NO) were higher in the rat ileum than the jejunum 34, 35. Also, the release of protective peptides following IR injury is significantly higher in the ileum when compared with the jejunum 36. It is interesting that the therapeutic vasculoprotective effects of MSCs were observed in the more damaged jejunum rather than the lesser injured ileum. This suggests circulating MSCs may require high concentrations of inflammatory mediators or chemical stress signals to be present locally before they “switch on” protective mechanisms. Interestingly, the occasional intravital images of MSCs appearing to “release” their cytoplasmic content within the injured mucosa were mostly captured in the jejunum. Adherent neutrophils can contribute to vascular congestion and no‐reflow—hence dampening the neutrophil infiltrate may explain the resumption of tissue perfusion and early improvements in jejunal blood flow. MSCs are also well known for their ability to secrete biologically significant amounts of NO, driven by inducible nitric oxide synthase (iNOS) 37. Previous studies have shown that upregulation of iNOS mRNA does not occur until around 2–4 hours post activation 37. If MSC‐derived NO is mediating improvements in vascular tone following IR injury, this effect would likely be observed at later time points, as seen in the ileum in this study.

It is interesting that despite poor MSC presence, neutrophil infiltration was modified. It may be possible that high levels of local MSC recruitment are not required in order to realize their therapeutic benefit. MSCs are potent anti‐inflammatory cells and as such the level of MSC recruitment required for therapeutic function may be met with the observed basal recruitment. Alternatively, the localization of MSCs may not be critically important for inducing therapeutic activity. There is growing experimental data supporting the idea that, after intravenous injection, MSCs interact with immunologic cells located in distant organs (primarily the lungs) thereby altering the systemic immunologic/inflammatory response. Indeed, MSCs located in the pulmonary microvasculature are able to secrete factors which improve outcomes in other tissues, such as the heart and the brain 38, 39, 40. Such results indicate that it may not be necessary for a large number of cells to reach the injured tissue to produce an effect.

We previously demonstrated that HSC pretreatment with soluble inflammatory factors enhanced their adhesion within IR injured gut. Such pretreatments modified adhesion either by enhancing integrin clustering on HSCs and/or increasing their affinity/avidity for endothelial counterligands. Cytokine treatment of MSCs has also been shown previously to upregulate adhesion molecule expression on their surface 41. However, in this study, these pretreatment strategies did not improve MSC adhesion. This suggests MSC recruitment may not be an active process or reflect poor upregulation of adhesion molecules on PDGFRα^+^ murine MSCs. In light of our data suggesting MSC recruitment is mediated primarily by physical entrapment, we postulated that our chemical prestimulations would improve adhesion through effects on deformability or other physical characteristics that may influence cell entrapment. Indeed, HSC deformability increases with factors such as CXCL12, preventing nonspecific entrapment in sites such as the lungs and thus maintaining a larger pool of circulating cells in the peripheral blood 42. We did not note changes in recruitment in vivo following stimulation of cells with these treatments. It is known that oxidative stress (H_2_O_2_) can lead to increased cell stiffness of MSCs 4. This may exacerbate the problem of cell entrapment and thus explain the lack of enhanced adhesion in the gut. Indeed, in this study, H_2_O_2_ pretreatment was noted to increase MSC presence, albeit nonsignificantly, in the lungs.

Interestingly, it is possible that MSC recruitment in vivo is inhibited in platelet‐featuring pathologies. Platelets play an important pathological role in many ischemic disorders. Indeed, following intestinal IR injury platelet recruitment begins as early as 5 minutes post‐reperfusion 43. Recent work by Vogel et al. demonstrated that conditioned media derived from activated platelets strongly inhibited MSC migration towards injured cardiomyocytes in vitro 44. However, our static adhesion assays also showed no increase in MSC adhesion to platelet‐free, immobilized endothelial ligands ICAM‐1, VCAM‐1, and MAdCAM‐1 following any pretreatment. In addition, MSC adhesion was not enhanced to activated endothelium either. Collectively, this data suggest that MSCs may be poorly adhesive and as such, any effects of stimulations before administration may not be sufficient to enhance their recruitment when administered in vivo.

Although no modification of MSC adhesion was observed, preexposure of MSCs to inflammatory mediators may potentiate the release of paracrine factors. Thus, pretreatment may render MSCs of greater therapeutic benefit. Indeed, evidence suggests that the immunosuppressive potency of MSCs is greatly increased when prestimulated with IFNγ 45. Furthermore, pretreatment with IL‐1β has been shown to enhance the therapeutic efficacy of MSC transplantation in a mouse model of colitis, when compared with naive cells 46. We first tested whether pretreatment could stimulate release of potentially beneficial anti‐inflammatory factors, namely IL‐10, IL‐13, and IL‐6, from primary PDGFR^+^ MSCs. Release of proinflammatory IL‐1β and TNFα, was also tested. Significant increases in IL‐6 were detected following pretreatment with TNFα and IFNγ. Cellular release of IL‐6 peaked at 2 hours post‐stimulation with decreased IL‐6 release detected thereafter. Although MSCs express the receptors TNFR1, TNFR2, and IFNγR1, our data suggest that these receptors may be engaged in activities that modulate cytokine release rather than the adhesive capabilities of MSCs. The potential importance of IL‐6 as a beneficial paracrine factor released from MSCs is given significance in light of evidence that it can limit warm hepatic IR injury through down regulation of TNFα release 47. IL‐6 has also been shown to drive release of secondary mediators such as prostaglandin E2 (PGE_2_) 48. Furthermore, exogenously administered IL‐6 has also been shown to protect the inner retina after IR injury 49. Future studies could address the possibility of administering IL‐6 as an adjuvant to maximise the efficacy cellular therapy.

As TNFα and IFNγ were most effective at stimulating IL‐6 release from MSCs at 2 hours, we further tested for enhanced therapeutic efficacy of these pretreated cells in vivo. Again, improvements in mucosal blood flow and down regulation of neutrophil adhesion were investigated compared with vehicle treated MSCs. MSCs were stimulated with TNFα or IFNγ for 1 hour and then systemically introduced into IR injured mice. Surprisingly, pretreated cells failed to confer the vasculoprotective effects previously observed by naive MSCs in the jejunum. However, in contrast, previously nontherapeutic MSCs decreased ileal neutrophil adhesion when IFNγ treated. Collectively this suggests pretreatment abolishes the MSC vasculoprotective effects in areas of severe tissue injury, but may render them vasculoprotective in regions of limited tissue injury. The undermining of previously beneficial MSCs in the jejunum may be due to a shift towards earlier “peak release” of paracrine mediators. Maximal IL‐6 release was noted at 2 hours post‐stimulation yet IR injury becomes progressively worse with time. It is also possible that IFNγ (but not TNFα) may cause the release of an unknown factor that is able to reduce neutrophil recruitment in the lesser injured ileum. Alternatively, given that less IL‐6 was secreted in vitro with IFNγ compared with TNFα, MSCs may not have been “depleted” to the same degree before having a chance to confer an anti‐inflammatory action in the ileum. Clearly a central role for MSC‐derived IL‐6 is apparent as it has been demonstrated in a number of studies to limit local release of proinflammatory mediators. In a model of carbon tetrachloride (CCl_4_) induced hepatic injury, evidence suggests IL‐6 plays an important role in ameliorating hepatic injury by MSCs 50. In a model of LPS‐induced pulmonary injury, IL‐6 mediates the protective effects of adipose derived MSCs (ASCs) 51.

## Conclusion

In conclusion, our data show that limited MSCs home successfully to the injured gut mucosa, an event that we could not improve. However, despite this, MSCs were vasculoprotective in that they were able to downregulate neutrophil adhesion and improve blood flow. For the first time, we show that the severity of injury, even in the same organ, impacted on the therapeutic efficacy of MSCs. Furthermore, stimulation of MSCs before administration may not always be beneficial and may in some scenarios hinder the ability of these cells to perform their anti‐inflammatory functions. With the number of clinical trials involving MSCs increasing, this current data suggest that pretreatment strategies should be carefully considered and validated before use. Although there is an urgency to identify strategies that promote MSC recruitment to sites of injury, it is equally important to identify and rule out those strategies that do may negatively impact on their therapeutic potential. In this study, cytokine pretreatment presents itself as a double‐edged sword whereby the benefits in the lesser injured regions of the gut may be offset by loss of benefit in the severely injured gut.

## Author Contributions

D.P.J.K.: designed and performed experiments, analyzed data, and drafted the manuscript; S.S.: performed experiments and proofed the manuscript; P.N.N. and J.F.: provided reagents and proofed the manuscript; N.K.: obtained funding, analyzed data, designed experiments, and drafted the manuscript.

## Disclosure of Potential Conflicts of Interest

The authors indicate no potential conflicts of interest.

## References

[1] Houlihan DD , Newsome PN . Critical review of clinical trials of bone marrow stem cells in liver disease. Gastroenterology 2008;135:438–450. 18585384 10.1053/j.gastro.2008.05.040

[2] Kavanagh DPJ , Robinson J , Kalia N . Mesenchymal stem cell priming: Fine‐tuning adhesion and function. Stem Cell Rev Rep 2014;10:587–599. 10.1007/s12015-014-9510-7 24752328

[3] Rüster B , Göttig S , Ludwig RJ et al. Mesenchymal stem cells display coordinated rolling and adhesion behavior on endothelial cells. Blood 2006;108:3938–3944. 16896152 10.1182/blood-2006-05-025098

[4] Toma C , Wagner WR , Bowry S et al. Fate of culture‐expanded mesenchymal stem cells in the microvasculature: In vivo observations of cell kinetics. Circ Res 2009;104:398–402. 19096027 10.1161/CIRCRESAHA.108.187724 PMC3700384

[5] Teo GS , Yang Z , Carman CV et al. Intravital imaging of mesenchymal stem cell trafficking and association with platelets and neutrophils. Stem Cells 2015;33:265–277. 25263183 10.1002/stem.1848 PMC4270897

[6] Kavanagh DP , Yemm AI , Alexander JS et al. Enhancing the adhesion of hematopoietic precursor cell integrins with hydrogen peroxide increases recruitment within murine gut. Cell Transp 2013;22:1485–1499. 10.3727/096368912X653192 22889470

[7] Kavanagh DP , Yemm AI , Zhao Y et al. Mechanisms of adhesion and subsequent actions of a haematopoietic stem cell line, HPC‐7, in the injured murine intestinal microcirculation in vivo. PLoS One 2013;8:e59150. 23554986 10.1371/journal.pone.0059150 PMC3595270

[8] Swenson E , Theise N . Stem cell therapeutics: Potential in the treatment of inflammatory bowel disease. Clin Exp Gastroenterol 2010;3:1–10. 21694840 PMC3108654

[9] Gonzalez MA , Gonzalez‐Rey E , Rico L et al. Adipose‐derived mesenchymal stem cells alleviate experimental colitis by inhibiting inflammatory and autoimmune responses. Gastroenterology 2009;136:978–989. 19135996 10.1053/j.gastro.2008.11.041

[10] Parekkadan B , Tilles AW , Yarmush ML . Bone marrow‐derived mesenchymal stem cells ameliorate autoimmune enteropathy independently of regulatory T cells. Stem Cells 2008;26:1913–1919. 18420833 10.1634/stemcells.2007-0790

[11] Dalal J , Gandy K , Domen J . Role of mesenchymal stem cell therapy in Crohn's disease. Pediatr Res 2012;71:445–451. 22430380 10.1038/pr.2011.56

[12] Zhang J , Chen GH , Wang YW et al. Hydrogen peroxide preconditioning enhances the therapeutic efficacy of Wharton's Jelly mesenchymal stem cells after myocardial infarction. Chin Med J (Engl) 2012;125:3472–3478. 23044308

[13] Kim YS , Park HJ , Hong MH et al. TNF‐alpha enhances engraftment of mesenchymal stem cells into infarcted myocardium. Front Biosci 2009;14:2845–2856. 10.2741/3417 19273239

[14] Kwon YW , Heo SC , Jeong GO et al. Tumor necrosis factor‐α‐activated mesenchymal stem cells promote endothelial progenitor cell homing and angiogenesis. Biochim Biophys Acta 2013;1832:2136–2144. 23959047 10.1016/j.bbadis.2013.08.002

[15] Sivanathan KN , Gronthos S , Rojas‐Canales D et al. Interferon‐gamma modification of mesenchymal stem cells: Implications of autologous and allogeneic mesenchymal stem cell therapy in allotransplantation. Stem Cell Rev 2014;10:351–375. 24510581 10.1007/s12015-014-9495-2

[16] Sorkine P , Setton A , Halpern P et al. Soluble tumor necrosis factor receptors reduce bowel ischemia‐induced lung permeability and neutrophil sequestration. Crit Care Med 1995;23:1377–1381. 7634808 10.1097/00003246-199508000-00011

[17] Souza AL, Jr. , Poggetti RS , Fontes B et al. Gut ischemia/reperfusion activates lung macrophages for tumor necrosis factor and hydrogen peroxide production. J Trauma 2000;49:232–236. 10963533 10.1097/00005373-200008000-00008

[18] Wu MCL , Brennan FH , Lynch JPL et al. The receptor for complement component C3a mediates protection from intestinal ischemia‐reperfusion injuries by inhibiting neutrophil mobilization. Proc Natl Acad Sci 2013;110:9439–9444. 23696668 10.1073/pnas.1218815110 PMC3677481

[19] Braun F , Hosseini M , Wieland E et al. Kinetics and localization of interleukin‐2, interleukin‐6, heat shock protein 70, and interferon gamma during intestinal‐rerfusion injury. Transpl Proc 2004;36:267–269. 10.1016/j.transproceed.2004.01.082 15050130

[20] Keating A . Mesenchymal stromal cells. Curr Opin Hematol 2006;13:419–425. 17053453 10.1097/01.moh.0000245697.54887.6f PMC3365862

[21] Chang P , Qu Y , Liu Y et al. Multi‐therapeutic effects of human adipose‐derived mesenchymal stem cells on radiation‐induced intestinal injury. Cell Death Dis 2013;4:e685. 23788042 10.1038/cddis.2013.178 PMC3698545

[22] Eggenhofer E , Benseler V , Kroemer A et al. Mesenchymal stem cells are short‐lived and do not migrate beyond the lungs after intravenous infusion. Front Immunol 2012;3:297. 23056000 10.3389/fimmu.2012.00297 PMC3458305

[23] Houlihan DD , Mabuchi Y , Morikawa S et al. Isolation of mouse mesenchymal stem cells on the basis of expression of Sca‐1 and PDGFR‐alpha. Nat Protoc 2012;7:2103–2111. 23154782 10.1038/nprot.2012.125

[24] Yipp BG , Kubes P . Antibodies against neutrophil LY6G do not inhibit leukocyte recruitment in mice in vivo. Blood 2013;121:241–242. 23287627 10.1182/blood-2012-09-454348

[25] Ando T , Jordan P , Wang Y et al. MAdCAM‐1 expression and regulation in murine colonic endothelial cells in vitro. Inflam Bowel Dis 2005;11:258–264. 10.1097/01.mib.0000160807.53858.1c 15735432

[26] Holyer I , Watson SP , Kalia N . PC62 (Conference Proceedings) The role of platelet GPIIb and PLC[gamma]2 in murine intestinal ischemia‐reperfusion (IR) injury in vivo. Microcirculation 2009;16:444–486.

[27] Kavanagh DP , Durant LE , Crosby HA et al. Haematopoietic stem cell recruitment to injured murine liver sinusoids depends on (alpha)4(beta)1 integrin/VCAM‐1 interactions. Gut 2010;59:79–87. 19828466 10.1136/gut.2008.168054

[28] Hoogduijn MJ , Beukel JCvd , Wiersma LCM et al. Morphology and size of stem cells from mouse and whale: Observational study. BMJ 2013;347:f6833. 10.1136/bmj.f6833 PMC3898169 24336001

[29] Rostgaard J , Qvortrup K . Electron microscopic demonstrations of filamentous molecular sieve plugs in capillary fenestrae. Microvasc Res 1997;53:1–13. 9056471 10.1006/mvre.1996.1987

[30] Aldridge V , Garg A , Davies N et al. Human mesenchymal stem cells are recruited to injured liver in a β1‐integrin and CD44 dependent manner. Hepatology 2012;56:1063–1073. 22422467 10.1002/hep.25716

[31] Thin Luu N , McGettrick HM , Buckley CD et al. Crosstalk between mesenchymal stem cells and endothelial cells leads to downregulation of cytokine‐induced leukocyte recruitment. Stem Cells 2013;31:2690–2702. 23939932 10.1002/stem.1511

[32] Kaminski PM , Proctor KG . Attenuation of no‐reflow phenomenon, neutrophil activation, and reperfusion injury in intestinal microcirculation by topical adenosine. Circ Res 1989;65:426–435. 2665971 10.1161/01.res.65.2.426

[33] Wagner JE , Collins D , Fuller S et al. Isolation of small, primitive human hematopoietic stem cells: Distribution of cell surface cytokine receptors and growth in SCID‐Hu mice. Blood 1995;86:512–523. 7541665

[34] Chan KL , Zhang XH , Fung CW et al. Role of nitric oxide in intestinal ischaemia‐reperfusion injury studied using electron paramagnetic resonance. Br J Surg 2002;86:1427–1432. 10583290 10.1046/j.1365-2168.1999.01241.x

[35] Chan KL , Chan KW , Tam PKH . Segmental small bowel allograft—Ischemic injury and regeneration. J Pediatr Surg 1998;33:1703–1706. 9856900 10.1016/s0022-3468(98)90614-5

[36] Kozar RA , Santora RJ , Poindexter BJ et al. Alterations in content and localization of defensins in rat ileum and jejunum following ischemia‐reperfusion. Specific peptides, in specific places, for specific jobs? Eplasty 2011;11:e8. 21369366 PMC3044598

[37] Ren G , Zhang L , Zhao X et al. Mesenchymal stem cell‐mediated immunosuppression occurs via concerted action of chemokines and nitric oxide. Cell Stem Cell 2008;2:141–150. 18371435 10.1016/j.stem.2007.11.014

[38] Lee RH , Pulin AA , Seo MJ et al. Intravenous hMSCs improve myocardial infarction in mice because cells embolized in lung are activated to secrete the anti‐inflammatory protein TSG‐6. Cell Stem Cell 2009;5:54–63. 19570514 10.1016/j.stem.2009.05.003 PMC4154377

[39] Choi H , Lee RH , Bazhanov N et al. Anti‐inflammatory protein TSG‐6 secreted by activated MSCs attenuates zymosan‐induced mouse peritonitis by decreasing TLR2/NF‐kappaB signaling in resident macrophages. Blood 2011;118:330–338. 21551236 10.1182/blood-2010-12-327353 PMC3138686

[40] Roddy GW , Oh JY , Lee RH et al. Action at a distance: Systemically administered adult stem/progenitor cells (MSCs) reduce inflammatory damage to the cornea without engraftment and primarily by secretion of TNF‐α stimulated gene/protein 6. Stem Cells 2011;29:1572–1579. 21837654 10.1002/stem.708

[41] Ren G , Zhao X , Zhang L et al. Inflammatory cytokine‐induced intercellular adhesion molecule‐1 and vascular cell adhesion molecule‐1 in mesenchymal stem cells are critical for immunosuppression. J Immunol 2010;184:2321–2328. 20130212 10.4049/jimmunol.0902023 PMC2881946

[42] White RL , Nash G , Kavanagh DP et al. Modulating the adhesion of haematopoietic stem cells with chemokines to enhance their recruitment to the ischaemically injured murine kidney. PLoS ONE 2013;8:e66489. 23840488 10.1371/journal.pone.0066489 PMC3686749

[43] Massberg S , Enders G , Leiderer R et al. Platelet‐endothelial cell interactions during ischemia/reperfusion: The role of P‐selectin. Blood 1998;92:507–515. 9657750

[44] Vogel S , Chatterjee M , Metzger K et al. Activated platelets interfere with recruitment of mesenchymal stem cells to apoptotic cardiac cells via high mobility group box 1/toll‐like receptor 4‐mediated down‐regulation of hepatocyte growth factor receptor MET. J Biol Chem 2014;289:11068–11082. 24567328 10.1074/jbc.M113.530287 PMC4036247

[45] Krampera M , Cosmi L , Angeli R et al. Role for interferon‐gamma in the immunomodulatory activity of human bone marrow mesenchymal stem cells. Stem Cells 2006;24:386–398. 16123384 10.1634/stemcells.2005-0008

[46] Fan H , Zhao G , Liu L et al. Pre‐treatment with IL‐1beta enhances the efficacy of MSC transplantation in DSS‐induced colitis. Cell Mol Immunol 2012;9:473–481. 23085948 10.1038/cmi.2012.40 PMC4002219

[47] Camargo CA, Jr. , Madden JF , Gao W et al. Interleukin‐6 protects liver against warm ischemia/reperfusion injury and promotes hepatocyte proliferation in the rodent. Hepatology 1997;26:1513–1520. 9397992 10.1002/hep.510260619

[48] Bouffi C , Bony C , Courties G et al. IL‐6‐dependent PGE2 secretion by mesenchymal stem cells inhibits local inflammation in experimental arthritis. PLoS One 2010;5:e14247. 21151872 10.1371/journal.pone.0014247 PMC2998425

[49] Sanchez RN , Chan CK , Garg S et al. Interleukin‐6 in retinal ischemia reperfusion injury in rats. Invest Ophthalmol Vis Sci 2003;44:4006–4011. 12939322 10.1167/iovs.03-0040

[50] Nasir GA , Mohsin S , Khan M et al. Mesenchymal stem cells and Interleukin‐6 attenuate liver fibrosis in mice. J Transl Med 2013;11:78. 23531302 10.1186/1479-5876-11-78 PMC3636128

[51] Zhang S , Danchuk SD , Bonvillain RW et al. Interleukin 6 mediates the therapeutic effects of adipose‐derived stromal/stem cells in lipopolysaccharide‐induced acute lung injury. Stem Cells 2014;32:1616–1628. 24449042 10.1002/stem.1632 PMC4365913

